# Defining, Evaluating, and Managing Postoperative Respiratory Depression: An e-Delphi Study

**DOI:** 10.7759/cureus.104612

**Published:** 2026-03-03

**Authors:** Sabry Ayad, Claudio S Pergolizzi, Robert B Raffa, Roshna Noor, Joseph V Pergolizzi

**Affiliations:** 1 Outcomes Research, Cleveland Clinic, Cleveland, USA; 2 Anesthesiology, Cleveland Clinic, Cleveland, USA; 3 Anesthesiology, Cleveland Clinic Fairview Hospital, Cleveland, USA; 4 Clinical Research, NEMA Research, Naples, USA; 5 Pharmacology, Enalare Therapeutics, New Jersey, USA; 6 Research and Development, Enalare Therapeutics, New Jersey, USA

**Keywords:** anesthesia, capnography, delphi method, expert consensus, opioid-induced respiratory depression, pain management, patient safety, perioperative monitoring, postoperative pulmonary complications, postoperative respiratory depression

## Abstract

Background: Postoperative respiratory depression (PORD) is a common but underreported postoperative pulmonary complication with potentially serious short- and long-term consequences. Despite its prevalence, no universally accepted expert definition exists, making identification, diagnosis, and epidemiologic study challenging.

Methods: An e‑Delphi study was conducted with a multidisciplinary panel of 10 board‑certified physicians in anesthesiology, surgery, pulmonology, emergency medicine, and critical care. Over four rounds, panelists anonymously rated 20 literature‑derived statements on PORD definition, diagnosis, risk factors, monitoring, prevention, and management using a 1-10 Likert scale. A consensus threshold of ≥70% agreement was applied. Panelists could suggest revisions, and statements were ranked by relative importance.

Results: Nineteen of 20 statements (95%) achieved consensus; 15 reached consensus within two rounds, and four additional statements reached consensus by round four. The most important statement defined PORD as a clinically significant reduction in neural drive to breathe, indicated by respiratory rate <10 breaths/min, SpO₂ <90%, or PaCO₂ >50-55 mmHg within 48 h post-surgery, with early cases often linked to anesthesia. Key consensus areas included risk factors (advanced age, obesity, pre-existing respiratory disease, certain anesthetic agents), diagnosis via continuous pulse oximetry and capnography, and the need for a reversal agent that does not compromise analgesia. A statement on low-dose naloxone infusion did not achieve consensus.

Conclusions: This study establishes expert consensus with a consensus threshold of ≥70% agreement on a clinically applicable definition of PORD, its risk factors, diagnostic criteria, and management principles. The findings provide a framework for standardizing research, guiding perioperative monitoring, and informing preventive strategies, while highlighting the urgent need for targeted reversal agents.

## Introduction

The Lancet Commission on Global Surgery estimates that at least 313 million surgical procedures are performed worldwide each year, most of them in developed nations [1]. Postoperative respiratory complications following major surgery affect approximately 1%-23% of patients, with postoperative respiratory depression (PORD) representing the most common type of complication [2,3]. Despite its prevalence, there is no universally accepted expert definition of PORD, making its identification, epidemiologic study, and the development of evidence-based guidelines challenging.

Because postoperative respiratory complications often resolve within 24-48 hours, PORD may be erroneously dismissed as a minor or self-limited issue, similar to what has previously been described for postoperative nausea and vomiting [4]. In reality, PORD is far more than a benign postoperative event. Broadly defined postoperative pulmonary complications are associated with substantial short- and long-term mortality. Patients experiencing pulmonary complications within 30 days of surgery have markedly higher mortality rates compared with those without complications (20% vs. 3% at 30 days, 24% vs. 1% at 90 days, 46% vs. 9% at one year, and 71% vs. 41% at five years) [5]. The specific contribution of PORD to these outcomes remains unclear.

Failure to accurately identify and promptly treat PORD in the hospital setting can result in catastrophic consequences, including cardiorespiratory arrest, anoxic brain injury, and death. Despite this risk, no universally accepted guidelines exist to define, diagnose, or manage PORD. The Agency for Healthcare Research and Quality has identified postoperative respiratory failure as the fourth most common patient safety event [5]. Without a clinically actionable definition and expert guidance, it is impossible to accurately quantify the burden of PORD or develop targeted prevention and treatment strategies. Although both patient-related and surgery-related risk factors have been proposed, including the type of anesthesia used, these factors have not been consistently defined or operationalized [5].

The goal of this electronic Delphi (e-Delphi) study was to establish expert consensus on the prevalence and clinical impact of PORD, define PORD in a clinically meaningful manner, and identify best practices for its identification, monitoring, prevention, and management.

## Materials and methods

The primary objective of the study was to reach a consensus on the key definitions, diagnostics, and management strategies for PORD. In addition, the study sought to establish the perceived prevalence and impact of PORD, establish PORD-related practices, and explore strategies to prevent and treat PORD. Secondary objectives sought to collect participant-related data and professional experiences, conduct bias analysis, and explore how these approaches align with the Enhanced Recovery After Surgery and Anesthesia Patient Safety Foundation guidance. An expert panel of 10 physicians was sought to provide an evaluable sample. Panelists were board-certified physicians from acute-care specialties in current clinical practice for at least the last five years and who have been published on relevant topics in the body of peer-reviewed literature. The expert panelists were blinded to each other’s identities to prevent bias, and their responses were anonymized prior to analysis. The logistics of the survey were managed by an administrator who was not blinded but who did not participate in data analysis, statement refinement, or the preparation of the manuscript.

Statements were to be developed based on a review of the peer-reviewed literature conducted by the study facilitator. The main areas of interest were current PORD definitions, risk factors, diagnostic criteria, and PORD management strategies. These statements were to be reviewed and revised, as needed, by the Principal Investigator and would compose round 1. Four rounds of voting were permitted. This four-round e-Delphi study structure described here adheres to best practices for consensus methodology [6,7].

Panelists were to vote anonymously and electronically using the Welphi software platform (Welphi, Lisbon, Portugal). Panelists were asked to rank each statement 1 to 10 using a Likert scale, where 10 represents total agreement. Panelists were also allowed to suggest revisions to statements and offer comments. Consensus was achieved if at least 70% of the panelists rated a statement ≥7. If a statement did not reach 70% consensus after four rounds, it was excluded from the consensus statements. If a critical statement failed to achieve consensus after four rounds, the Principal Investigator could initiate a follow-up survey to discuss the issue. The cutoff value for consensus for Delphi studies can be set by each study, but there is agreement that 70% is a valid threshold [8].

The Likert scale provided ordinal data, and median scores and percentages of agreement were calculated to evaluate consensus. Categorical variables, such as consensus levels, are reported as frequencies and percentages. Continuous variables, such as ranking, refinements of statements, and open-ended responses, were analyzed to identify trends. Qualitative data in the form of comments were used to refine statements, address ambiguities, and enhance the iterative process.

If a statement achieved consensus in two rounds, it was removed from the survey and further iteration.

The statements were also to be ranked by the panelists in order of their relative importance to the other statements in the survey. Relative importance was evaluated using an interquartile range (IQR) of rankings for each statement for each round. The use of an IQR value identified outliers. The average IQR over four rounds was then used to define the relative importance of each statement.

To reduce bias and preserve anonymity, which is essential to an e-Delphi study, each panelist was to be assigned a unique identification code. All survey responses were submitted individually and with no direct communication among panelists. Panelists were anonymous and were not aware of the identities of the others on the panel. Surveys were supervised by an unblinded study administrator who managed attendance tracking, logistics, and handled technical support. The study administrator played no role in data analysis, statement refinement, or the content of this article.

The study did not require any protected health information (PHI) to be disclosed. The study administrator was tasked with reviewing survey results and being alert to any potential inadvertent disclosure of PHI. Should any PHI be revealed in the survey, the study administrator was to censor it.

This study was reviewed by the NEMA Independent Ethics Committee, which issued an exemption determination. Voluntary participation in the Welphi platform implied consent.

## Results

A total of 25 candidates were screened as experts for the panel, of whom 11 were enrolled. One of the 11 panelists discontinued the study before the first round; ten panelists completed the study and met the requirement to provide an evaluable sample. The panelists consisted of multidisciplinary specialists in the fields of anesthesiology, pulmonology, surgery, emergency medicine, and critical care (Table 1).

**Table 1:** All panelists were board-certified physicians with a minimum of five years of clinical experience in acute-care specialties and a history of peer-reviewed publications relevant to postoperative respiratory depression (PORD). Several panelists held leadership positions in anesthesiology, surgery, critical care, and pulmonary medicine. Publications refer to published peer-reviewed articles in literature related to PORD and related topics.

Panelist ID	Specialty	Years in Practice	Board Certified	Academic Affiliation	Publications
P1	Anesthesiology, Critical Care	10+	Yes (Anes & CCM)	Wake Forest Univ. School of Medicine	Yes
P2	Anesthesiology	25+	Yes	MD Anderson Cancer Center	Yes
P3	Anesthesiology	25+	Yes	Stanford University	Yes
P4	Surgery	30+	Yes	Cleveland Clinic Florida	Yes
P5	Pulmonary, Sleep, COPD	30+	Yes	University of Pittsburgh	Yes
P6	Emergency Medicine	~9	Yes	Magnolia Regional Health Center	Yes
P7	Pulmonary, Critical Care, Sleep	30+	Yes	Tufts University	Yes
P8	Anesthesiology	20+	Yes	Augusta University / Ohio State	Yes
P9	Anesthesiology, Critical Care	5+	Yes (Anes & CCM)	Mayo Clinic	Yes
P10	Anesthesiology	25+	Yes	University of Utah	Yes

The study commenced on November 26, 2024, and concluded on January 10, 2025, taking four rounds to complete. Twenty statements were used in the survey. The statements were developed based on a comprehensive review of the literature addressing postoperative respiratory depression, postoperative pulmonary complications, monitoring strategies, analgesic approaches, and risk prediction models [1-43]. Mean Likert scores with corresponding standard deviations are reported for each statement, alongside the proportion of panelists achieving consensus (rating ≥7).

Panelists emphasized that monitoring recommendations apply across common postoperative care environments, including the post-anesthesia care unit (PACU) and subsequent inpatient locations (ward, step-down, and ICU as clinically indicated). The highest risk interval was considered the early postoperative period, particularly the first 12-24 hours, with ongoing risk extending up to 48 hours depending on patient factors, opioid exposure, and clinical course.

The finalized Delphi statements, Likert scores, and supporting references are presented in Table 2.

**Table 2:** Delphi statements and supporting literature. The 20 statements from the expert panel with supporting literature are summarized here. References are described by the surname of the first author and year published. A complete reference list is available in the reference section.

Statement	Mean Likert Score ± SD	Consensus (%)	Supporting References
Definition of PORD: Postoperative respiratory depression (PORD) is a clinically significant reduction in the neural drive for breathing. Signs of PORD include respiratory rate below 10 breaths per minute, oxygen saturation (SpO₂) below 90%, or elevated carbon dioxide levels (PaCO2 above 50-55 mmHg) occurring within 48 hours post-surgery.	9.3 ± 0.8	100%	Ko et al., 2003 [9]
Criteria for Diagnosing PORD: Diagnosis of PORD should utilize a combination of continuous pulse oximetry, capnography, and regular clinical assessments to monitor respiratory rate, oxygen saturation, and carbon dioxide levels.	9.0 ± 1.0	100%	Khanna et al., 2024 [10]
Risk Factors for PORD: PORD can be attributed to various risk factors across different stages of patient care. Intraoperative factors include the use of general anesthesia and prolonged surgery duration. Perioperative risk factors include advanced age and obesity, and dexmedetomidine use. Postoperative risk factors encompass pre-existing respiratory conditions, such as COPD, sleep apnea, and postoperative opioid use and certain medications (neuromuscular blockers, benzodiazepines).	8.8 ± 1.1	100%	Albrecht et al., 2020 [31], Bevilacqua et al., 2021 [29]
Impact of PORD on Postoperative Outcomes: Patients experiencing PORD have an increased risk of 30- and 90-day morbidity and mortality in the post-hospital setting, with long-term consequences extending beyond the immediate postoperative period.	8.7 ± 1.0	100%	Miskovic et al., 2017 [2]
Management of PORD: Management strategies for PORD should include preoperative optimization, intraoperative protective ventilation, and postoperative monitoring with interventions such as supplemental oxygen, ventilatory support (HFNC, BIPAP, intubation) and opioid antagonists as needed	7.9 ± 1.7	90%	Luo et al., 2024 [30]
Preventive Measures for PORD: Preventive measures include implementing multimodal analgesia to reduce opioid consumption, optimizing patient positioning to enhance respiratory function, and integrating Enhanced Recovery After Surgery (ERAS) protocols. This approach reduces PORD incidence by improving pain management and respiratory function.	8.6 ± 1.0	100%	Verma et al., 2021 [11]
Monitoring for PORD: Continuous monitoring of respiratory function using pulse oximetry and capnography is essential in the early postoperative period to detect and manage PORD effectively.	8.4 ± 1.2	100%	Ayad et al., 2019 [5]
Rise of Noninvasive Ventilation (NV) in PORD: Prophylactic noninvasive ventilation (NIV) has not been shown to significantly reduce the incidence of postoperative acute respiratory failure in high-risk patients, primarily due to low patient compliance. However, NIV may be effective as a rescue therapy when PORD is already present, as compliance issues are less significant in this context and its efficacy depends on the reduction of ventilatory drive.	8.1 ± 1.1	100%	Abrard et al., 2023 [12]
Intrathecal Morphine and PORD: Intrathecal morphine, while effective for postoperative analgesia, can cause respiratory depression. This effect is particularly concerning in patients with pre-existing conditions, such as sleep apnea (OSA) or other respiratory conditions, where the risk of PORD may be heightened.	7.8 ± 1.5	90%	Dhaliwal et al., 2019 [13]
Naloxone for PORD: Low-dose naloxone infusion can reduce the incidence of respiratory depression in patients receiving intrathecal morphine without significantly compromising pain control.	6.0 ± 2.0	N/A	Cosgrave et al., 2020 [14]
Intrathecal Analgesia and PORD: Intrathecal administration of morphine and bupivacaine provide effective early postoperative analgesia and reduces the requirement for additional IV opioids in patients undergoing surgery.	8.3 ± 1.0	100%	Dereu et al., 2019 [15], Shim et al., 2021 [16]
Multimodal Analgesia and Continuous Infusion Multimodal analgesic strategies, including continuous infusion of analgesics like methadone and dexmedetomidine, can provide sustained sedation and pain control with minimal risk of respiratory depression. Careful monitoring is required.	7.7 ± 1.6	90%	Amer et al., 2020 [17] Morue et al., 2018 [18] Ranganathan et al., 2019 [19] Sadhasivam et al., 2021 [20]
Postoperative Pain Management and PORD: Effective pain management strategies must balance adequate analgesia with minimizing the risk of respiratory depression, particularly in high-risk patients.	8.2 ± 1.0	100%	Comelon et al., 2021 [21] Han et al., 2018 [22] He et al., 2021 [23] Shanthanna et al., 2019 [24]
Education on PORD: Patients and healthcare providers should receive continuous education on the detection, monitoring, and management of PORD. Awareness of risk factors and compliance with preventive measures is critical to improving patient outcomes.	8.0 ± 1.2	100%	Tarasova et al., 2024 [25] Assadi et al., 2025 [26]
Future Research Direction in PORD: Future research should focus on developing and validating predictive models for PORD, identifying new preventive strategies, and improving multimodal analgesic protocols to minimize the risk of respiratory depression.	8.1 ± 1.1	100%	Ayad et al., 2019 [5]
Impact of PORD on Lung and Systemic Inflammation: PORD prolongs atelectasis and ventilator-related heterogeneity in lung inflation, resulting in an inflammatory cascade that can propagate damage other organ systems outside of the lung.	7.8 ± 1.5	90%	Lusquinhos et al., 2023 [28]
Continuous Pulse Oximetry (CPOX) and Capnography for PORD Prevention: Continuous pulse oximetry (CPOX), with or without capnography, significantly enhances the early detection of postoperative hypoxemia (SpO₂ < 90%) compared to routine intermittent monitoring. Despite this, no significant differences were found in ICU transfer rates, reintubation, noninvasive ventilation, or length of stay. Implementing CPOX may improve early recognition of PORD, although its impact on more severe outcomes remains unclear.	8.5 ± 1.0	100%	Khanna et al., 2024 [10] Lam et al., 2017 [27]
Need for PORD Reversal Agent: There is a need for a PORD reversal agent that does not impact anesthesia or analgesia. Naloxone can reverse respiratory depression, but it also increases pain, highlighting the need for more targeted solutions	8.7 ± 0.9	100%	Cosgrave et al., 2020 [14]
Prediction of Opioid-Induced Respiratory Depression (OIRD): Key predictors of OIRD include age ≥ 60, male sex, opioid naivete, sleep disorders, and chronic heart failure. Continuous monitoring with capnography and oximetry enhances early detection and can reduce the risk of adverse outcomes.	8.2 ± 1.0	100%	Urman et al., 2021 [33]
Monitoring Limitation in PORD: Current monitoring techniques, such as respiratory rate and pulse oximetry, often fail to detect early signs of respiratory depression, particularly in opioid-treated patients. Advanced multimodal systems combining capnography and pulse oximetry may significantly improve detection and patient safety.	8.0 ± 1.1	100%	Lam et al., 2017 [27]

Terminology relating to PORD, postoperative opioid-induced respiratory depression (POIRD), and postoperative pulmonary complications is sometimes used imprecisely, but there is some overlap in these conditions. POIRD, as the name implies, is the result of residual opioids in the system or what might be commonly called “oversedation” [32,33]. PORD is a broader term that encompasses respiratory depression not necessarily caused by opioids (although opioids may be a cause or contributor to PORD); nonopioid causes of PORD include residual anesthetics in the patient (such as propofol), inadequate reversal of neuromuscular blocking agents, sedating medications other than opioids (such as benzodiazepines or gabapentinoids), advanced age, pre-existing pulmonary disease, and various other patient-related factors (such as obstructive sleep apnea and obesity). Postoperative pulmonary complications might also include atelectasis and hypoventilation, which could be brought on by prolonged duration of surgery or certain types of thoracic surgeries that restrict normal respiration [34]. These overlapping terms have caused confusion, particularly when it comes to gathering epidemiologic data and finding effective treatments, which was a compelling reason for this study [2]. Overall, PORD is a broader term than POIRD, but POIRD is a specific subset of PORD. The experts were aware that these terms are sometimes used imprecisely in the literature.

Consensus was achieved in the first two rounds for 75% of the statements (15/20). Of the remaining five statements, four achieved consensus by the fourth round of voting. One statement failed to achieve consensus. A summary of results appears in Table 3.

**Table 3:** Statements were presented to panelists in the original order specified (far left column), and panelists could rank them based on their relative importance (rank). If there was relevant discussion or modifications to the statement, these discussions are summarized in the column for expert feedback with the final consensus statement after that. Changes or additions made to the original statement are bolded and italicized in the final statement. The final column reports the number of rounds (minimum 2, maximum 4) required to achieve consensus. All statements except #10 achieved consensus.

Original statement	Rank	Expert feedback	Final statement	Rounds
Definition of PORD: Postoperative respiratory depression (PORD) is a clinically significant reduction in the neural drive for breathing. Signs of PORD include respiratory rate below 10 breaths per minute, oxygen saturation (SpO₂ below 90%, or elevated carbon dioxide levels (PaCO2 above 50-55 mmHg) occurring within 48 hours post-surgery	1	Early vs. Late PORD Distinguish early PORD (≤24 hours), which is more likely linked to anesthesia, from late PORD (>24 hours), which is less likely related to intraoperative factors. Reduce respiratory rate threshold to 8 breaths per minute.	Definition of PORD: Postoperative respiratory depression (PORD) is a clinically significant reduction in the neural drive for breathing. Signs of PORD include respiratory rate below 10 breaths per minute, oxygen saturation (SpO₂) below 90%, or elevated carbon dioxide levels (PaCO2 above 50-55 mmHg) occurring within 48 hours post-surgery, with cases within the first 24 hours often linked to anesthesia-related factors	2
Criteria for Diagnosing PORD: Diagnosis of PORD should utilize a combination of continuous pulse oximetry, capnography, and regular clinical assessments to monitor respiratory rate, oxygen saturation, and carbon dioxide levels.	3	None	No change	2
Risk Factors for PORD: PORD can be attributed to various risk factors across different stages of patient care. Intraoperative factors include the use of general anesthesia and prolonged surgery duration. Perioperative risk factors include advanced age and obesity, and dexmedetomidine use. Postoperative risk factors encompass pre-existing respiratory conditions, such as COPD, sleep apnea, and postoperative opioid use and certain medications (neuromuscular blockers, benzodiazepines).	2	Reconsider including dexmedetomidine as a risk factor. While dexmedetomidine can cause sedation, it typically does not affect respiratory drive correlating to respiratory depression.	Risk Factors for PORD: PORD can be attributed to various risk factors across different stages of patient care. Intraoperative factors include the use of general anesthesia and prolonged surgery duration. Perioperative risk factors include advanced age and obesity. Postoperative risk factors encompass pre-existing respiratory conditions, such as COPD, sleep apnea, and postoperative opioid use and certain medications (neuromuscular blockers, benzodiazepines).	2
Impact of PORD on Postoperative Outcomes: Patients experiencing PORD have an increased risk of 30- and 90-day morbidity and mortality in the post-hospital setting, with long-term consequences extending beyond the immediate postoperative period.	5	None	No change	2
Management of PORD: Management strategies for PORD should include preoperative optimization, intraoperative protective ventilation, and postoperative monitoring with interventions such as supplemental oxygen, ventilatory support (HFNC, BIPAP, intubation) and opioid antagonists as needed	9	Add continuous postoperative monitoring and multimodal analgesia.	Management of PORD: Management strategies for PORD should include preoperative optimization, including weight loss, smoking cessation, and weaning off opioids; intraoperative protective ventilation (with recruitment breaths as appropriate for patients with diminished functional reserve capacity undergoing prolonged procedures), and continuous postoperative monitoring with interventions such as supplemental oxygen, ventilatory support (HFNC, BIPAP, intubation), multimodal analgesia, and opioid antagonists as needed	4
Preventive Measures for PORD: Preventive measures include implementing multimodal analgesia to reduce opioid consumption, optimizing patient positioning to enhance respiratory function, and integrating Enhanced Recovery After Surgery (ERAS) protocols. This approach reduces PORD incidence by improving pain management and respiratory function.	14	Consider patients with opioid use disorder, where tailored strategies and education are necessary.	No change	2
Monitoring for PORD: Continuous monitoring of respiratory function using pulse oximetry and capnography is essential in the early postoperative period to detect and manage PORD effectively.	17	Capnography requires sophisticated monitors that may not be available in all postoperative settings.	No change	2
Rise of Noninvasive Ventilation (NV) in PORD: Prophylactic noninvasive ventilation (NIV) has not been shown to significantly reduce the incidence of postoperative acute respiratory failure in high-risk patients, primarily due to low patient compliance. However, NIV may be effective as a rescue therapy when PORD is already present, as compliance issues are less significant in this context and its efficacy depends on the reduction of ventilatory drive.	10	The effectiveness of NIV in treating PORD depends on the choice of device and settings, which should be customized to suit patients.	No change	2
Intrathecal Morphine and PORD: Intrathecal morphine, while effective for postoperative analgesia, can cause respiratory depression. This effect is particularly concerning in patients with pre-existing conditions, such as sleep apnea (OSA) or other respiratory conditions, where the risk of PORD may be heightened.	12	Whether intrathecal morphine is commonly used in patients with obstructive sleep apnea (OSA) or not, the side effects of additional sedatives should also be taken into account.	Intrathecal Morphine and PORD: While intrathecal morphine, while effective is used for postoperative analgesia, it can cause increase respiratory depression. This effect is particularly concerning in patients with pre-existing conditions, such as sleep apnea (OSA) or other respiratory conditions, or when co-administered with other drugs that have sedative side effects, where the risk of PORD may be heightened.	4
Naloxone for PORD: Low-dose naloxone infusion can reduce the incidence of respiratory depression in patients receiving intrathecal morphine without significantly compromising pain control.	20	Concerns about the limitations of this therapy.	No consensus	No consensus
Intrathecal Analgesia and PORD: Intrathecal administration of morphine and bupivacaine provide effective early postoperative analgesia and reduces the requirement for additional IV opioids in patients undergoing surgery.	15	Reservations regarding the actual benefits and the limited duration of local anesthetics.	Intrathecal Analgesia and PORD: Intrathecal administration of opioids (such as morphine) and local anesthetics (such as bupivacaine) provide effective early postoperative analgesia and reduces the requirement for additional IV opioids in patients undergoing surgery.	2
Multimodal Analgesia and Continuous Infusion Multimodal analgesic strategies, including continuous infusion of analgesics like methadone and dexmedetomidine, can provide sustained sedation and pain control with minimal risk of respiratory depression. Careful monitoring is required.	8	Reservations about the feasibility of using epidural infusions in routine practice, particularly due to the need for extensive monitoring. Methadone and dexmedetomidine are not mainstream multimodal techniques.	Multimodal Analgesia and Continuous Infusion: Multimodal analgesic strategies, including continuous epidural infusions of local anesthetics when appropriate, may enhance postoperative pain control with minimal risk of respiratory depression. These strategies should be implemented with structured monitoring and combined with nonopioid analgesics to optimize safety and effectiveness.	4
Postoperative Pain Management and PORD: Effective pain management strategies must balance adequate analgesia with minimizing the risk of respiratory depression, particularly in high-risk patients.	16	Clearer definition of high-risk patients	No change	2
	18	None	No change	2
Future Research Direction in PORD: Future research should focus on developing and validating predictive models for PORD, identifying new preventive strategies, and improving multimodal analgesic protocols to minimize the risk of respiratory depression.	19	New pharmacotherapy for reducing the risk of PORD.	No change	2
Impact of PORD on Lung and Systemic Inflammation: PORD prolongs atelectasis and ventilator-related heterogeneity in lung inflation, resulting in an inflammatory cascade that can propagate to damage other organ systems outside of the lung.	6	This is an important area for education, particularly in the context of patients who require intubation with mechanical ventilation or NIV significant pressures.	Impact of PORD on Lung and Systemic Inflammation: PORD prolongs atelectasis and ventilator-related heterogeneity in lung inflation, resulting in an inflammatory cascade that can propagate to damage other organ systems outside of the lung. This is particularly relevant for postoperative patients requiring intubation with mechanical ventilation or noninvasive ventilation (NIV) at significant pressures.	4
Continuous Pulse Oximetry (CPOX) and Capnography for PORD Prevention: Continuous pulse oximetry (CPOX), with or without capnography, significantly enhances the early detection of postoperative hypoxemia (SpO₂ < 90%) compared to routine intermittent monitoring. Despite this, no significant differences were found in ICU transfer rates, reintubation, noninvasive ventilation, or length of stay. Implementing CPOX may improve early recognition of PORD, although its impact on more severe outcomes remains unclear.	7	None	Continuous Pulse Oximetry and Capnography for PORD Prevention: Continuous pulse oximetry (CPOX), with or without capnography, significantly enhances the early detection of postoperative hypoxemia (SpO₂ < 90%) compared to routine intermittent monitoring. Despite this, no significant differences were found in ICU transfer rates, reintubation, noninvasive ventilation, or length of stay. Implementing CPOX may improve early recognition of PORD, although its impact on more severe outcomes remains unclear. Evidence is limited by the heterogeneity of studies and the lack of clinical trials with a control group.	2
Need for PORD Reversal Agent: There is a need for a PORD reversal agent that does not impact anesthesia or analgesia. Naloxone can reverse respiratory depression, but it also increases pain, highlighting the need for more targeted solutions	4	None	No change	2
Prediction of Opioid-Induced Respiratory Depression (OIRD): Key predictors of OIRD include age ≥ 60, male sex, opioid naivete, sleep disorders, and chronic heart failure. Continuous monitoring with capnography and oximetry enhances early detection and can reduce the risk of adverse outcomes.	11	State sleep apnea specifically rather than “sleep disorders.” Obesity is a predictor.	Prediction of Opioid-Induced Respiratory Depression (OIRD): Key predictors of OIRD derived from continuous pulse oximetry and capnography episodes include age ≥ 60, male sex, opioid naivete, sleep apnea disorders, obesity, and chronic heart failure. Continuous monitoring with capnography and oximetry enhances early detection and can reduce the risk of adverse outcomes.	2
Monitoring Limitation in PORD: Current monitoring techniques, such as respiratory rate and pulse oximetry, often fail to detect early signs of respiratory depression, particularly in opioid-treated patients. Advanced multimodal systems combining capnography and pulse oximetry may significantly improve detection and patient safety.	13	None	No change	2

Nineteen of the statements submitted to the expert panel achieved consensus, of which 15 reached consensus in two rounds. Discussion leading to revision occurred for eight of the statements. This suggests that there is a strong consensus among experts as to the prevalence, diagnosis, treatment, and monitoring needed for PORD. The single statement that failed to reach consensus regarded the use of low-dose naloxone infusion to reduce PORD, yet there was agreement that a reversal agent for PORD is an unmet medical need. Four rounds of voting were taken on the naloxone statement, and the failure to meet the consensus threshold suggests that the reservations expressed by some respondents against naloxone were particularly strong.

Respondents were also asked to rank the statements in order of importance, shown in Table 4.

**Table 4:** The 19 statements on which the expert panel reached consensus in their final form are ranked in order of relative importance, with 1 being most important.

Rank	Final statement
1	Definition of PORD: Postoperative respiratory depression (PORD) is a clinically significant reduction in the neural drive for breathing. Signs of PORD include respiratory rate below 10 breaths per minute, oxygen saturation (SpO₂) below 90%, or elevated carbon dioxide levels (PaCO2 above 50-55 mmHg) occurring within 48 hours post-surgery, with cases within the first 24 hours often linked to anesthesia-related factors.
2	Risk Factors for PORD: PORD can be attributed to various risk factors across different stages of patient care. Intraoperative factors include the use of general anesthesia and prolonged surgery duration. Perioperative risk factors include advanced age and obesity. Postoperative risk factors encompass pre-existing respiratory conditions, such as COPD, sleep apnea, and postoperative opioid use and certain medications (neuromuscular blockers, benzodiazepines).
3	Criteria for Diagnosing PORD: Diagnosis of PORD should utilize a combination of continuous pulse oximetry, capnography, and regular clinical assessments to monitor respiratory rate, oxygen saturation, and carbon dioxide levels.
4	Need for PORD Reversal Agent: There is a need for a PORD reversal agent that does not impact anesthesia or analgesia. Naloxone can reverse respiratory depression, but it also increases pain, highlighting the need for more targeted solutions.
5	Impact of PORD on Postoperative Outcomes: Patients experiencing PORD have an increased risk of 30- and 90-day morbidity and mortality in the post-hospital setting, with long-term consequences extending beyond the immediate postoperative period.
6	Impact of PORD on Lung and Systemic Inflammation: PORD prolongs atelectasis and ventilator-related heterogeneity in lung inflation, resulting in an inflammatory cascade that can propagate to damage other organ systems outside of the lung. This is particularly relevant for postoperative patients requiring intubation with mechanical ventilation or noninvasive ventilation (NIV) at significant pressures.
7	Continuous Pulse Oximetry and Capnography for PORD Prevention: Continuous pulse oximetry (CPOX), with or without capnography, significantly enhances the early detection of postoperative hypoxemia (SpO₂ < 90%) compared to routine intermittent monitoring. Despite this, no significant differences were found in ICU transfer rates, reintubation, noninvasive ventilation, or length of stay. Implementing CPOX may improve early recognition of PORD, although its impact on more severe outcomes remains unclear. Evidence is limited by the heterogeneity of studies and the lack of clinical trials with a control group.
8	Multimodal Analgesia and Continuous Infusion: Multimodal analgesic strategies, including continuous epidural infusions of local anesthetics when appropriate, may enhance postoperative pain control with minimal risk of respiratory depression. These strategies should be implemented with structured monitoring and combined with nonopioid analgesics to optimize safety and effectiveness.
9	Management of PORD: Management strategies for PORD should include preoperative optimization, including weight loss, smoking cessation, and weaning off opioids; intraoperative protective ventilation (with recruitment breaths as appropriate for patients with diminished functional reserve capacity undergoing prolonged procedures), and continuous postoperative monitoring with interventions such as supplemental oxygen, ventilatory support (HFNC, BIPAP, intubation), multimodal analgesia, and opioid antagonists as needed.
10	Rise of Noninvasive Ventilation (NV) in PORD: Prophylactic noninvasive ventilation (NIV) has not been shown to significantly reduce the incidence of postoperative acute respiratory failure in high-risk patients, primarily due to low patient compliance. However, NIV may be effective as a rescue therapy when PORD is already present, as compliance issues are less significant in this context and its efficacy depends on the reduction of ventilatory drive.
11	Prediction of Opioid-Induced Respiratory Depression (OIRD): Key predictors of OIRD derived from continuous pulse oximetry and capnography episodes include age ≥ 60, male sex, opioid naivete, sleep apnea, obesity, and chronic heart failure. Continuous monitoring with capnography and oximetry enhances early detection and can reduce the risk of adverse outcomes.
12	Intrathecal Morphine and PORD: While intrathecal morphine is used for postoperative analgesia, it can increase respiratory depression. This effect is particularly concerning in patients with pre-existing conditions, such as sleep apnea (OSA) or other respiratory conditions, or when co-administered with other drugs that have sedative side effects, where the risk of PORD may be heightened.
13	Monitoring Limitation in PORD: Current monitoring techniques, such as respiratory rate and pulse oximetry, often fail to detect early signs of respiratory depression, particularly in opioid-treated patients. Advanced multimodal systems combining capnography and pulse oximetry may significantly improve detection and patient safety.
14	Preventive Measures for PORD: Preventive measures include implementing multimodal analgesia to reduce opioid consumption, optimizing patient positioning to enhance respiratory function, and integrating Enhanced Recovery After Surgery (ERAS) protocols. This approach reduces PORD incidence by improving pain management and respiratory function.
15	Intrathecal Analgesia and PORD: Intrathecal administration of opioids (such as morphine) and local anesthetics (such as bupivacaine) provide effective early postoperative analgesia and reduces the requirement for additional IV opioids in patients undergoing surgery.
16	Postoperative Pain Management and PORD: Effective pain management strategies must balance adequate analgesia with minimizing the risk of respiratory depression, particularly in high-risk patients.
17	Monitoring for PORD: Continuous monitoring of respiratory function using pulse oximetry and capnography is essential in the early postoperative period to detect and manage PORD effectively.
18	Education on PORD: Patients and healthcare providers should receive continuous education on the detection, monitoring, and management of PORD. Awareness of risk factors and compliance with preventive measures is critical to improving patient outcomes.
19	Future Research Direction in PORD: Future research should focus on developing and validating predictive models for PORD, identifying new preventive strategies, and improving multimodal analgesic protocols to minimize the risk of respiratory depression.

Those items ranked with the highest relative importance are fundamental statements related to a definition of PORD, risk factors, diagnosis, and the unmet medical need for a reversal agent. The main concern raised in the comments with respect to naloxone is the potential of naloxone to interfere with analgesia. The failure of a statement on low-dose naloxone infusion to achieve consensus suggests that the majority of the expert panel rejects naloxone as that agent, not that such an agent is not needed.

Of primary importance in this study was the expert consensus definition of PORD with clinically relevant diagnostic criteria. This statement reached consensus in two rounds and was unanimously voted first in relative importance. The consensus statements indicate that PORD is prevalent, frequently encountered in clinical practice, and may have effects on postoperative outcomes. The key cutoff values and parameters needed for a clinical definition of PORD achieved consensus. Among the multidisciplinary panelists, there was an emphasis on avoiding PORD to the greatest extent possible and suggesting future research directions that may involve noninvasive ventilation in PORD, alternative ventilation support, and pharmacologic interventions.

## Discussion

In general, this study found robust consensus. Seventy-five percent of the statements presented to the panel achieved consensus within two rounds of voting, indicating broad expert agreement on key topics related to PORD. Because this was a consensus-based study, the findings reflect expert interpretation of the existing literature rather than the generation of new quantitative outcome estimates.

The panel agreed that a clinically actionable definition of PORD is needed and reached consensus on threshold values (respiratory rate <10 breaths per minute, SpO₂ <90%, and PaCO₂ >50-55 mmHg) that are commonly used in clinical practice to identify respiratory compromise. While prior studies have reported associations between postoperative respiratory complications and adverse outcomes, the present findings represent expert consensus regarding the clinical relevance of these parameters rather than causal or quantitative effect estimates. Collectively, the consensus supports the view that PORD is a prevalent and clinically meaningful condition that can be identified using conventional, widely available clinical metrics.

Panelists emphasized that continuous monitoring should be tailored to the postoperative environment and local resources. Recommendations were considered applicable across common postoperative care settings, including the post-anesthesia care unit (PACU), inpatient wards, step-down units, and intensive care units, as clinically indicated. The highest-risk interval was viewed as the early postoperative period, particularly the first 12-24 hours, with extended monitoring up to 48 hours for patients with ongoing risk factors or continued opioid exposure.

When capnography is not feasible, panelists agreed that continuous pulse oximetry combined with frequent structured clinical assessments, including sedation level, respiratory rate, and work of breathing, represents the minimum acceptable monitoring approach. Escalation was recommended for sustained hypoxemia (e.g., SpO₂ <90% despite supplemental oxygen), bradypnea (e.g., respiratory rate <10 breaths/min), increasing sedation, or signs of clinical deterioration.

The goal of a consensus definition for PORD is to better inform multiple perioperative care pathways, including integrating it into Enhanced Recovery After Surgery (ERAS) protocols. Adding this PORD definition into surgical safety checklists and other screening criteria would assist the anesthesia team and other clinicians in identifying those at elevated risk for PORD and ensuring continuous monitoring and prompt intervention. Such action would be similar to the Surgical Safety Checklist published by the World Health Organization (WHO) [35]. Such criteria could be incorporated into monitoring alerts in electronic health records and other systems used to support clinical decision-making. Alert messages that are triggered when thresholds are reached (such as respiratory rate < 10/min, SpO₂ < 90%, PaCO₂ > 50-55 mmHg) would facilitate prompt intervention and care escalation strategies [36].

Four statements required four rounds of voting, indicating that for 20% of the topics, expert consensus was achievable with some revisions, because initial differences of opinion on these statements could be resolved with discussion. In particular, management strategies for PORD were a topic where the panel suggested expanding the scope of the work, adding the putative risk factor of obesity.

Only one of the 20 statements failed to achieve consensus, and it related to the possible suitability of low-dose naloxone as a reversal agent in the context of PORD. The main upshot of objections to low-dose naloxone infusion was the possibility that even at low doses, naloxone can compromise analgesia [37]. While naloxone is well recognized for its rapid and effective reversal of opioid toxicity [38], opioids in the perioperative setting cannot be reversed without compromising pain control. The optimal agent for PORD would be an agent that drives respiration at the neural level rather than at the level of the affected opioid receptors [39-41]. This relates to the statement panelists ranked as fourth in relative importance: the unmet need for an effective and safe respiratory driver to treat PORD.

As our population ages and surgeries increase globally, the need for better strategies related to PORD will likely be pushed to the forefront. PORD and other respiratory complications of surgery can place financial and resource burdens on the healthcare system. PORD is a common postoperative respiratory complication, which can result in long-term morbidity and mortality consequences [2]. The long-range consequences specific to PORD are not yet elucidated, but must cause a re-evaluation in terms of how the healthcare system manages PORD [42]. The definition is a major first step that can help experts better draft diagnostic criteria, establish risk factors, and offer guidelines specific to PORD [39,40].

For many decades, PORD has eluded a standard, objective definition and clear, universally accepted diagnostic criteria. We may want to consider why such a definition has been so difficult. Guidelines describe postoperative respiratory complications in broad terms, and most clinicians adjust the familiar metrics of respiratory rate, peripheral oxygen saturation, and partial pressure of arterial carbon dioxide to fit the patient, that is, making adjustments for the patient’s age, overall fitness, comorbidities, and the nature and duration of the surgery. So while objective metrics exist and are widely used, these measurements are “personalized” in the clinic. Since PORD often seemingly resolved spontaneously, the need for a consensus definition of PORD did not emerge as an urgent, unmet medical need until the recognition of greater post-discharge morbidity and mortality, telehealth, AI, and patient monitoring systems disrupted healthcare. Telehealth in all of its forms hampers clinical care in the sense that the clinician and patient are not in the same room; this demands objective metrics rather than the older techniques of face-to-face encounters, leading to diagnoses. Further, demands for better epidemiologic assessments of health-related conditions necessitate objective definitions. As new treatments evolve, a consensus definition of PORD will help with accurate diagnoses, quantification, and the ability to better evaluate and compare treatments in clinical trials. Such a definition lends support to the goals of the Joint Commission’s National Patient Safety goals that mandate identification of high-risk patients, effective communication among caregivers, and prompt and effective intervention for patients whose conditions deteriorate [43]. A universally accepted expert consensus definition of PORD within these established frameworks could improve diagnostic consistency, enhance benchmarking efforts that span multiple institutions, and provide for more accurate epidemiologic data collection.

Our study has certain limitations. It was conducted using an e-Delphi design, which allowed panelists to participate remotely via a secure online platform rather than through in-person meetings. Although the effects of virtual participation on consensus development have not been fully characterized, the anonymity of this format is recognized to enhance freedom to dissent and reduce groupthink. The panel consisted of ten board-certified physicians with extensive clinical and academic expertise, a sample size that aligns with established standards for Delphi methodology in expert groups [6]. All panelists were based in the United States, and these views may not be generalizable to a global population. While this is a limitation, it is somewhat mitigated by the fact that the panelists enjoy international recognition as key opinion leaders. The consensus statements have not yet been validated in a clinical population, and future studies are planned to assess their real-world applicability. While the panel was multidisciplinary and composed of physicians with significant experience across anesthesiology, surgery, critical care, emergency medicine, and pulmonology, further studies are needed to evaluate applicability across diverse clinical settings. Note that this was a Delphi study, and no quantitative outcome modeling was done. This would be an important step for future endeavors, but our initial efforts focused on achieving a consensus definition.

Future investigations should prioritize prospective validation of the proposed consensus definition in multicenter surgical populations, implementation studies evaluating structured monitoring strategies based on these criteria, and comparative effectiveness trials of pharmacologic or device-based respiratory support strategies. Core outcomes should include the need for rescue ventilation or noninvasive ventilation, naloxone administration, ICU transfer, reintubation, mortality, and patient-reported pain outcomes.

## Conclusions

PORD is a prevalent condition with long-term consequences that may not be fully recognized. Despite the fact that PORD is frequently encountered clinically, a lack of a consensus definition for the condition has impeded diagnosis and treatment, as well as epidemiologic assessments and study of potential risk factors and their mitigation. This e-Delphi study found expert consensus on a serviceable definition of PORD and its treatment, but could not reach agreement on a statement related to the role of low-dose naloxone in treating PORD. Nevertheless, the expert panel did agree that there is an urgent unmet medical need for some form of pharmacologic respiratory driver to treat PORD.
